# Machine learning-based signature of necrosis-associated lncRNAs for prognostic and immunotherapy response prediction in cutaneous melanoma and tumor immune landscape characterization

**DOI:** 10.3389/fendo.2023.1180732

**Published:** 2023-05-09

**Authors:** Zhiwei Cui, Zhen Liang, Binyu Song, Yuhan Zhu, Guo Chen, Yanan Gu, Baoyan Liang, Jungang Ma, Baoqiang Song

**Affiliations:** ^1^ Department of Plastic and Reconstructive Surgery, Xijing Hospital, Fourth Military Medical University, Xi’an, China; ^2^ Department of Cancer Center, Daping Hospital, Army Medical University, Chongqing, China

**Keywords:** cutaneous melanoma (CM), necroptosis, long non-coding RNAs (lncRNAs), prognostic signature, tumor immune function

## Abstract

**Background:**

Cutaneous melanoma (CM) is one of the malignant tumors with a relative high lethality. Necroptosis is a novel programmed cell death that participates in anti-tumor immunity and tumor prognosis. Necroptosis has been found to play an important role in tumors like CM. However, the necroptosis-associated lncRNAs’ potential prognostic value in CM has not been identified.

**Methods:**

The RNA sequencing data collected from The Cancer Genome Atlas (TCGA) and Genotype-Tissue Expression Project (GTEx) was utilized to identify differentially expressed genes in CM. By using the univariate Cox regression analysis and machine learning LASSO algorithm, a prognostic risk model had been built depending on 5 necroptosis-associated lncRNAs and was verified by internal validation. The performance of this prognostic model was assessed by the receiver operating characteristic curves. A nomogram was constructed and verified by calibration. Furthermore, we also performed sub-group K-M analysis to explore the 5 lncRNAs’ expression in different clinical stages. Function enrichment had been analyzed by GSEA and ssGSEA. In addition, qRT-PCR was performed to verify the five lncRNAs’ expression level in CM cell line (A2058 and A375) and normal keratinocyte cell line (HaCaT).

**Results:**

We constructed a prognostic model based on five necroptosis-associated lncRNAs (AC245041.1, LINC00665, AC018553.1, LINC01871, and AC107464.3) and divided patients into high-risk group and low-risk group depending on risk scores. A predictive nomogram had been built to be a prognostic indicator to clinical factors. Functional enrichment analysis showed that immune functions had more relationship and immune checkpoints were more activated in low-risk group than that in high-risk group. Thus, the low-risk group would have a more sensitive response to immunotherapy.

**Conclusion:**

This risk score signature could be used to divide CM patients into low- and high-risk groups, and facilitate treatment strategy decision making that immunotherapy is more suitable for those in low-risk group, providing a new sight for CM prognostic evaluation.

## 1 Introduction

Cutaneous melanoma (CM) is considered to be a malignant tumor that develops from melanocytes. CM is the result of a genetic mutation caused by ultraviolet ray radiation (1). Though it is not a high-incidence disease, it has a relatively high lethality rate. Multiple studies have revealed that CM occurs for 4% of skin cancers but 75% of skin cancer-related mortality (2). CM is one of the most immunogenic carcinomas and hence has a substantial potential for a positive response to immunotherapy (3). However, due to the early metastases, selecting the proper treatment strategy is critical for CM. Therefore, early detection of CM and stratified risk assessment are essential for CM treatment (4).

Necroptosis is a type of controlled cell death that mimics both necrosis and apoptosis. Plasma membrane permeabilization occurs rapidly during necroptosis. In this process, the cell content is released and then subsequently exposed to a variety of cytokines, chemokines, and damage-associated molecular patterns. The immunogenic nature of necroptotic cancer cells and their capacity to effectively trigger anti-tumor immunity are both attributed to necroptosis, which is becoming increasingly recognized as being crucial in cancer (5, 6). For example, the decrease in the expression of necroptotic factors such as receptor-interacting protein kinase-3 leads to a worse prognosis in breast cancer (7, 8). Similarly, the decrease of necroptotic factors’ expression, such as receptor-interacting protein kinase-3 and mixed lineage kinase domain-like protein, leads to reduced overall survival (OS) (9).

Long non-coding RNAs (lncRNAs) are those having transcripts that are 200 nucleotides or longer. Multiple studies have shown that lncRNAs are involved in cancer. lncRNA-Gm31932 uses the miR-344d-3-5p/Prc1 axis, for example, to induce cell cycle arrest and differentiation in melanoma (10). LncRNA TINCR suppresses melanoma cell proliferation and invasion by regulating the miR-424-5p/LATS1 axis, and it also upregulates apoptosis (11). Based on the important role of lncRNA in melanoma, several prognostic signatures have been established, like lncRNAs which are associated with autophagy (12), ferroptosis (2), pyroptosis (13), etc.

To create a unique predictive signature, this work analyzed the relationship between necroptosis-associated lncRNAs and clinicopathological features, and immune infiltration of individuals with CM. Internal verification and gene enrichment analysis (GSEA) were used to assess the robustness of the model as well as the potential mechanisms, respectively.

## 2 Methods

### 2.1 Data collection

The Cancer Genome Atlas (TCGA, n=471) was used to acquire RNA transcriptome datasets and associated clinical information of CM, and a synthetic data matrix concerning healthy skin data was obtained from Genotype-Tissue Expression Project (GTEx, n=234). The data were merged and processed using the R “Limma” tool. Furthermore, the lncRNA expression values and survival rates for 471 CM patients were determined.

### 2.2 Analysis of necroptosis-associated genes

The 201 necroptosis-associated genes were obtained from GeneCards (https://www.genecards.org/) and other published studies (**Table S1**). The “Limma” R-package was used to distinguish differentially expressed genes (DEGs) related to necroptosis in normal and CM tissues using a False Discovery Rate (FDR) of < 0.05 and a |log2 fold change (FC) >1| threshold (14). To determine which genes belong to both DEGs and necroptosis-associated, a Venn diagram was constructed. To visually represent the expression level of overlapping genes, a volcano image and a heatmap were created. The “ggplot2” package was utilized to conduct the analysis of the Kyoto Encyclopedia of Genes and Genomes (KEGG), and Gene Ontology (GO).

### 2.3 Necroptosis-associated lncRNAs signature associated with prognostic significance

The screening criteria for identifying the necroptosis-associated lncRNAs in CM samples with expression values were the correlation coefficients |R| > 0.3 and *p* < 0.001. Furthermore, to determine the necroptosis-associated lncRNA predictive signature and to assess the relationships between overall survival (OS) and necroptosis-associated lncRNAs in CM, the “survival” R package was used to perform a univariate Cox regression (uni-Cox) analysis at a significance level of *p* < 0.001. Then, the R package “caret” was employed to randomly classify the CM samples into the training and testing cohorts.

To identify the most important necroptosis-associated lncRNA with CM patients, we performed LASSO-penalized Cox regression analysis by the “glmnet” R package. The selection of variables in the Cox model was performed using the lasso method, and a risk signature was generated using the “survminer” package in R. The following calculations were utilized to determine the risk score: Risk score = sum (each lncRNA’s expression × corresponding coefficient). Low-risk (LR) and high-risk (HR) groups of CM patients were defined using the median risk score in both the training and testing datasets. The clinical information for the entire set was shown in **Table 1**. Potential lncRNA expression in normal and CM tissues was plotted using a heat map generated using the “pheatmap” R package.

**Table 1:** Different clinicopathological features of the necrosis-associated risk subgroups in TCGA-SKCM.

Clinical variables	Total (N=360)	Risk-group		*p* value
		high(n=178)	low(n=182)	
**Gender**				0.177
Female	138	62	76	
Male	222	116	106	
**Age**				0.962
<65	231	114	117	
≥65	129	64	65	
**Stage**				0.200
High stage	168	77	91	
Low stage	192	101	91	
**T**				0.025
T1-T2	134	56	78	
T3-T4	226	122	104	
**N**				0.185
N1-N2	313	159	154	
N3-N4	47	19	28	
**M**				0.840
M0	343	170	173	
M1	17	8	9	

The relationship between candidate lncRNA and mRNA was visualized by the Cytoscape diagram. Furthermore, “gg alluvial” in the R package was employed to visualize the distribution of the 5 candidate genes in LR and HR groups. The Kaplan-Meier (K-M) survival analysis and the correlation between risk score and survival time were performed using the “survival” and “survminer” packages in R. 1-year, 3-year, and 5-year ROC analyses were conducted using the “timeROC” R-package.

The “survival” R-package was used to create a prediction model, which included univariate and multivariate independent prognostic studies to determine the correlation between clinical features, risk score, and patient OS. A heatmap was made to illustrate the distribution of clinical features and potential lncRNAs in LR and HR groups. ROC analysis on risk scores and clinical features was done with the “survival ROC” package.

### 2.4 Nomogram and calibration

Using the risk score, age, and T, N stage, the R-package “rms” was used to create a nomogram for 1-year, 3-year, and 5-year OS. Using a calibration chart and ROC curves, we analyzed the nomogram’s prognostic accuracy.

### 2.5 Function enrichment analyses

The predominant route genes were analyzed using GSEA. GSEA 4.1.0 was employed to conduct the analysis. The cutoffs for statistical significance were minimal (FDR<0.25 and *p*<0.05). Here, the GSVA software and ssGSEA were used to determine the infiltration scores of 16 immunological cells and the 13 immune-related pathway activities.

### 2.6 Cell culture

Human normal keratinocyte cell line (HaCaT) and human melanoma cell lines (A2058 and A375) were purchased from American Type Culture Collection (ATCC). HaCaT and A375 cell lines were cultured in DMEM (Dulbecco’s Modified Eagle Medium) (Gibco, Grand Island, NY) and A2058 cell line was cultured in DMEM/F-12 (Gibco, Grand Island, NY) and both were added 10% fetal bovine serum (Yeasen, Shanghai, China) at 37°C in an incubator with 5% CO2.

### 2.7 RNA extraction and quantitative real-time polymerase chain reaction

Total RNA of the three cell lines was extracted with TRIzol Reagent (Takara, Kusatsu, Japan). cDNA was synthesized with Hifair^®^ III 1st Strand cDNA Synthesis SuperMix (11141ES60, Yeasen) according to standard protocol. The cDNA was used as a template and lncRNA expression was quantified using SYBR Green Master Mix (11184ES08, Yeasen). The primer pairs were synthesized by Tsingke Biotechnology (Beijing, China), and the primer pairs are listed in **Table S2**. All samples were normalized to GAPDH, and the 2^−ΔΔCt^ method was used to evaluate relative expression levels.

### 2.8 Statistical analysis

R 4.0.2 version and Prism 5 were used to conduct all statistical analyses. Analysis of continuous data was conducted using the Wilcoxon test, while analysis of categorical data was conducted using the Chi-square or Fisher tests. The log-rank test and the K-M technique were used to compare the OS rates of patients in the HR and LR groups. The collected findings were also considered significant at the *p* < 0.05 level.

## 3 Results

### 3.1 Necroptosis-associated lncRNAs in CM patients and functional analyses

**Figure 1:**
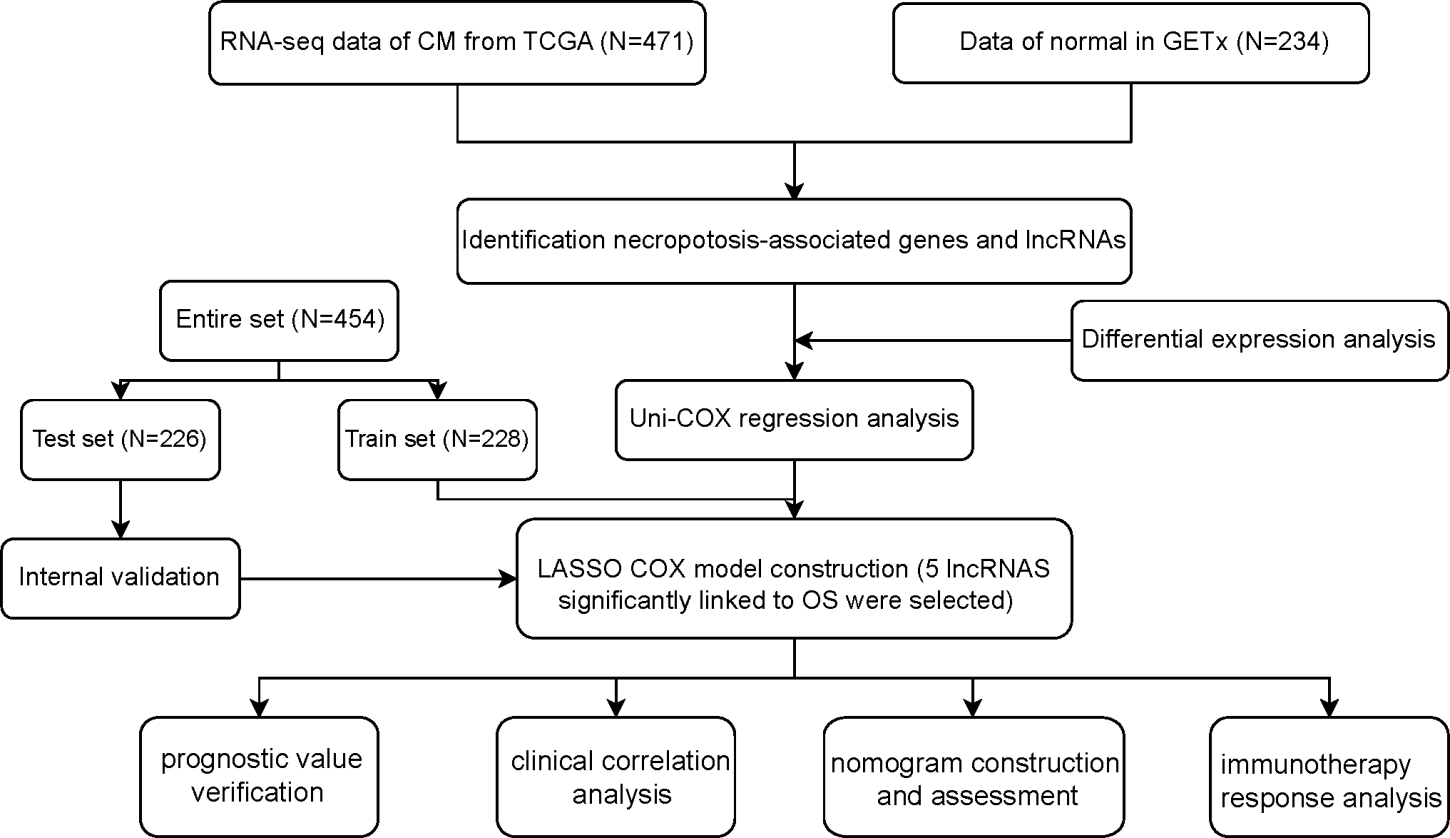
Flowchart of the study.

**Figure 2:**
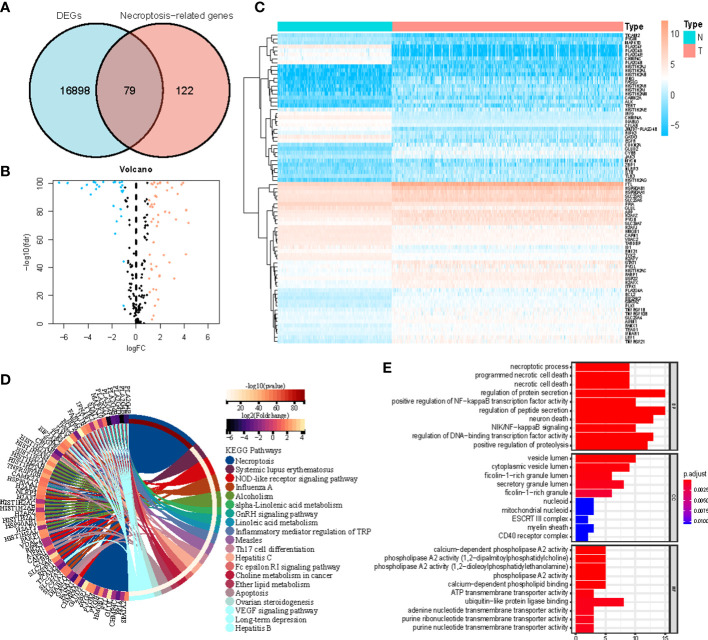
**(A)** Veen diagram of candidate necroptosis-associated differentially expressed genes (DEGs); **(B)** Volcano plot of 79 necroptosis-associated DEGs; **(C)** Heatmap visualizing the expression of necroptosis-associated DEGs; **(D, E)** KEGG and GO functional enrichment analysis of necroptosis-associated genes.

**Figure 1** shows how the study progressed. We got 234 normal samples and 471 CM samples from TCGA and GTEx. Finally, 79 predictive necroptosis-associated DEGs (correlation coefficients>0.3 and *p*<0.001) were identified by comparing the expression of 201 necroptosis-associated genes to 16,977 DEGs (|Log 2 FC|>1 and *p*<0.05) between normal and CM samples (**Figure 2A**). 52 of them had higher regulation, while the remaining 14 had lower regulation (**Figure 2B**). The heatmap was used to show the amount of ex-pression of the 79 overlapped genes in the normal and CM tissue (**Figure 2C**). The 79 overlapping genes were enriched in processes related to necroptosis, systemic lupus erythematosus, and NOD-like receptor signaling pathway, according to KEGG enrichment analysis (**Figure 2D**). The 79 overlapping genes were shown to be enriched in biological processes related to necroptotic and protein secretion, including the necroptotic process, programmed necrotic cell death, and control of protein and peptide secretion, according to GO enrichment analysis (**Figure 2E**).

### 3.2 Development of the necroptosis-associated lncRNA predictive signature

**Figure 3:**
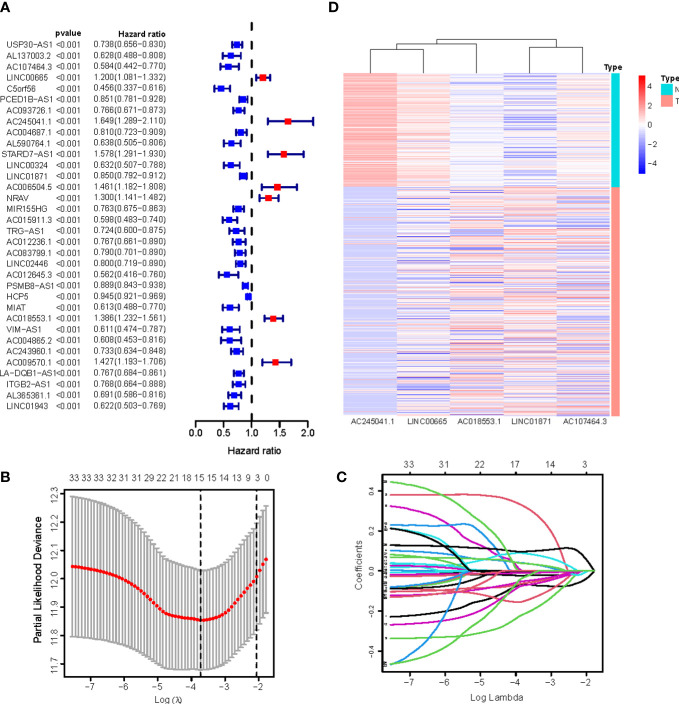
**(A)** 34 prognostic necroptosis-associated lncRNAs extracted by univariate Cox regression analysis; **(B)** The 10-fold cross-validation for variable selection in the LASSO model; **(C)** The LASSO coefficient profile of 16 necroptosis-associated lncRNAs; **(D)** Heatmap visualizing the expression of differentially expressed necroptosis-associated lncRNAs.

We identified 880 necroptosis-associated lncRNAs (**Table S3**). For the subsequent study, the expression levels of 880 lncRNAs associated with necroptosis and the clinical details of 454 melanoma samples were used. Using uni-Cox regression analysis, we identified 34 lncRNAs associated with necroptosis that were statistically significant predictors of OS (*p*<0.001) (**Figure 3A**). After running the Lasso regression on these lncRNAs, we found that 5 of them were associated with necroptosis in melanoma when the first-rank value of Log(λ) was the minimum likelihood of deviance, hence preventing the prognostic signature from overfitting (**Figures 3B, C**). The model contains the lncRNAs: AC245041.1, LINC00665, AC018553.1, LINC01871, and AC107464.3. The formula for the risk score is as follows: risk score = (-0.365383*expression of AC107464.3) + (0.26265*expression of LINC00665) + (0.56689*expression of AC245411.1) + (-0.45893*expression of LINC01871) + (0.48615*expression of AC018553.1). A heatmap depicts the differential expression of the five lncRNAs between normal and CM tissue (**Figure 3D**).

**Figure 4:**
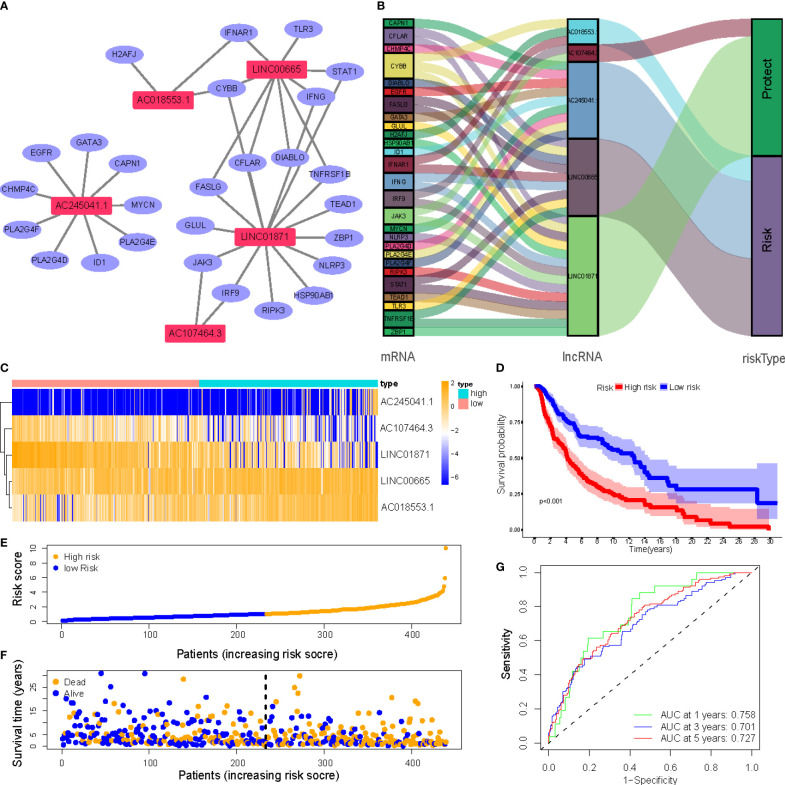
**(A)** Correlation network of prognostic lncRNAs and their associated genes; **(B)** The Sankey diagram of the prognostic lncRNAs and the related mRNAs; **(C)** Heatmap of 5 lncRNAs expression in the entire sets; **(D)** Kaplan–Meier (K-M) survival curves of OS of patients between low- and high-risk groups in the entire sets; **(E)** Exhibition of necroptosis-associated lncRNAs model based on risk score of the entire sets; **(F)** Survival time and survival status between low- and high-risk groups in the entire sets; **(G)** The 1-, 3-, and 5-year ROC curves of the entire sets.

**Figure 4A** depicts a network of the prognostic lncRNAs and the mRNAs they are linked to. Furthermore, this study identified AC107464.3 and LINC01871 as protective genes, whereas others were identified as risk factors (**Figure 4B**). It was also discovered that the LR and HR groups express the five lncRNAs in distinct ways (**Figure 4C**). LR and HR groups of CM patients were created using median risk scores (**Figures 4E, F**). When comparing the HR group to the LR group, **Figure 4D** shows that the HR group’s OS is significantly shorter (*p*<0.001). ROC study indicated that the risk signature had reasonable predictive accuracy at 1-year (ROC = 0.758), 2-year (ROC = 0.701), and 3-year (ROC = 0.727) (**Figure 4G**).

### 3.3 Independent prognostic factors and clinical correlation analysis of NALncSig

**Figure 5:**
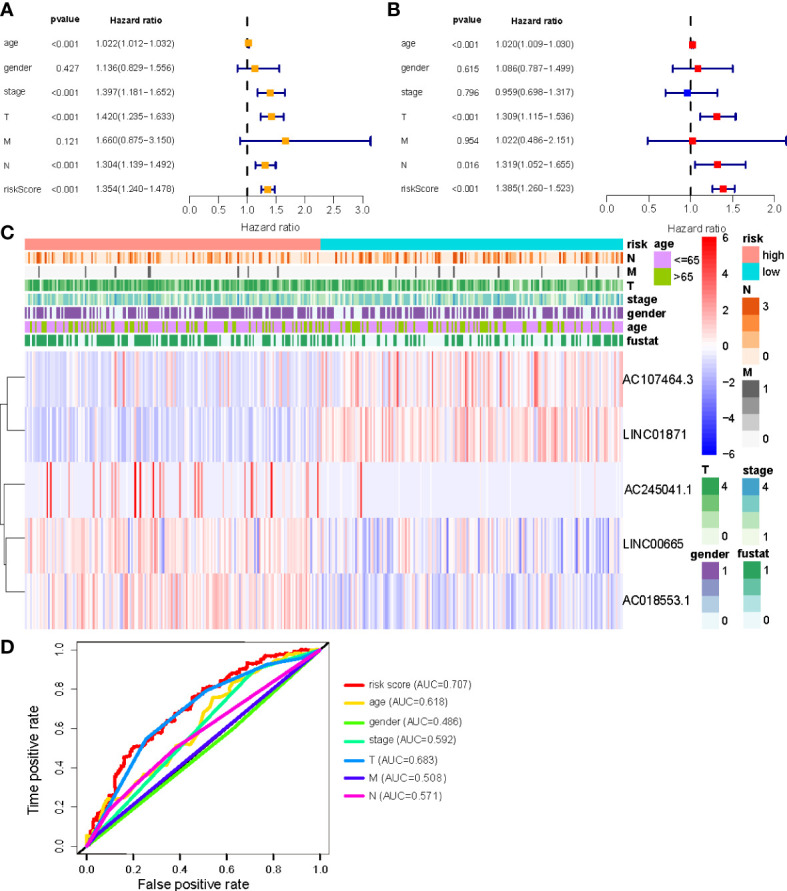
**(A, B)** Uni-Cox and Multi-Cox analyses of clinical factors and risk score with OS; **(C)** Heatmap of clinicopathological features and hub lncRNAs expression in two risk subgroups; **(D)** ClinicalROC curves to forecast overall survival of patients.

Univariate and multivariate Cox regression (multi-COX) analyses show that the newly identified risk signature is an independent prognostic factor for CM patients. The hazard ratio (HR) of the risk score and 95% confidence interval (CI) were 1.354 and 1.240-1.478 (*p*<0.001) in uni-Cox regression, respectively, 1.385 and 1.260-1.523 (*p*<0.001) in multi-Cox regression (**Figures 5A, B**). In addition, we found the other three independent prognostic parameters, age (1.020 and 1.009-1.030; *p*<0.001), T stage (1.309 and 1.115-1.536; *p*<0.001), and N stage (1.319 and 1.052-1.655; *p*=0.016) (**Figure 5B**). Our NALncSig was substantially correlated with age, T stage, and the N stage, according to the heatmap of clinical characteristics and risk groupings (**Figure 5C**). Additionally, various melanoma prognostic indicators were chosen for comparison to determine whether the NALncSig had the capacity for consistent and reliable performance. The ROC curve of the risk score and the clinicopathological criteria was performed. The area under the ROC curve (AUC) for our NALncSig curve was 0.707, which was significantly higher than the that for age (AUC = 0.618), gender (AUC = 0.486), stage (AUC = 0.592), T stage (AUC = 0.683), M stage (AUC = 0.508), and N stage (AUC = 0.571) (**Figure 5D**).

### 3.4 Construction of nomogram

**Figure 6:**
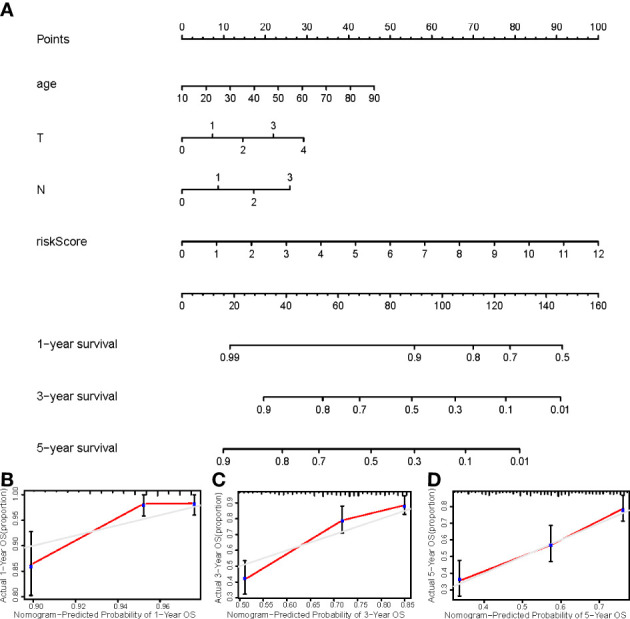
**(A)** The nomogram that integrated the risk score, age, and tumor stage predicted the probability of the 1-, 2-, and 3-year OS; **(B–D)** The calibration curves for 1-, 3-, and 5-year OS.

We constructed a nomogram using four independent prognostic factors risk score, age, T, and N (*p*<0.05 in multi-Cox) to predict the 1-, 3-, and 5-year OS incidences of CM patients (**Figure 6A**). Further confirmation of the nomogram’s accuracy in predicting these outcomes was obtained using 1-, 3-, and 5-year calibration plots (**Figures 6B–D**).

### 3.5 Relationship between the NALncSig and CM patient’s prognosis in different clinicopathological variables

**Figure 7:**
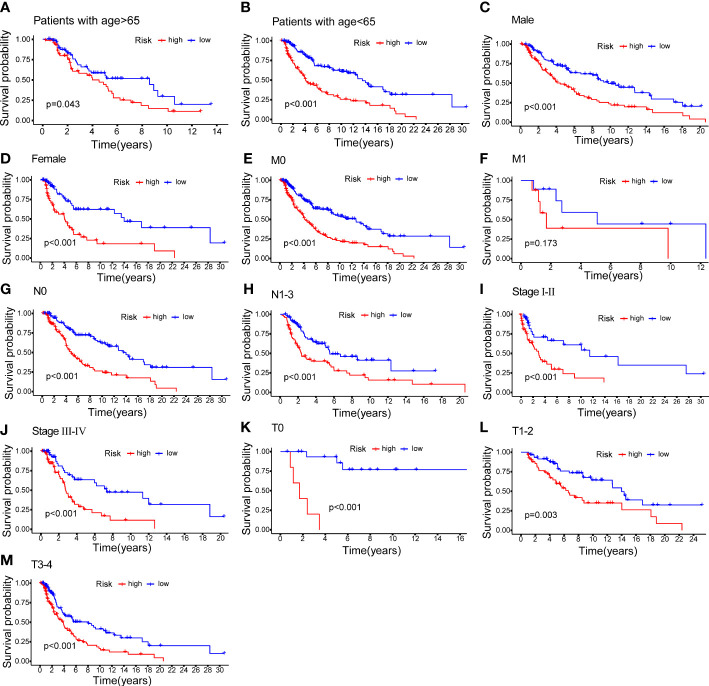
K–M methods for the two risk groups (high vs. low) categorized by clinical variables, comprising age **(A, B)**; gender **(C, D)**; M **(E, F)**; N **(G, H)**; stage **(I, J)** and T **(K–M)**.

**Figure 8:**
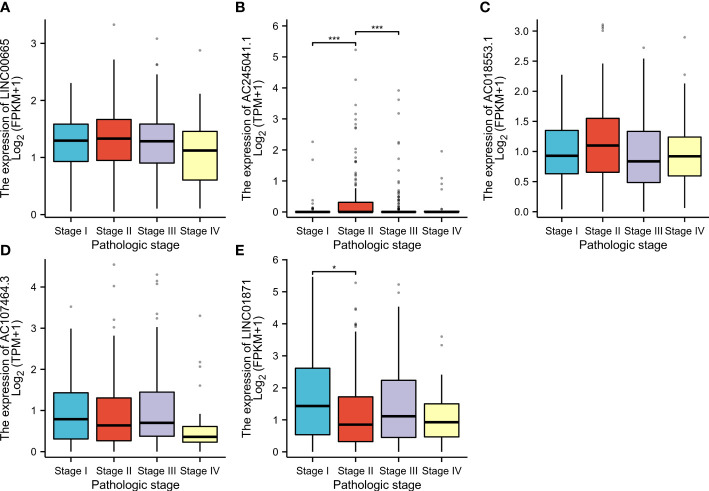
According to the clinical data, the relationship between pathologic stage and LINC00665 **(A)**; AC245041.1 **(B)**; AC245041.1 **(C)**; AC107464. 3 **(D)**; LINC01871 **(E)**. *p<0.05, ***p<0.001.

CM patients were divided into groups according to conventional clinicopathologic parameters such as age, gender, grade, and TNM stage. Except for patients with metastases (M1), the OS of patients in the HR groups was much lower than that of patients in the LR groups, demonstrating that the predictive signature can reliably predict the prognosis of CM patients (**Figures 7A-M**). Additionally, clinical data revealed that the expression of AC245041.1 had a difference between stage I and II, stage II and III (**Figure 8B**), and the expression of LINC01871 had a difference between stage I and II (**Figure 8E**), while the relationship between the expression of LNC00665, AC107464.3 and AC018553.1 and pathologic stage was not statistically significant(**Figures 8A, C, D**). These results indicate that various clinical variables have a certain effect on the expression and risk score of the necroptosis-associated lncRNA. Because of this, the pathological status of patients ought to be taken into consideration while developing the risk model that is used to evaluate the prognosis of patients.

### 3.6 Internal validation of NALncSig

**Figure 9:**
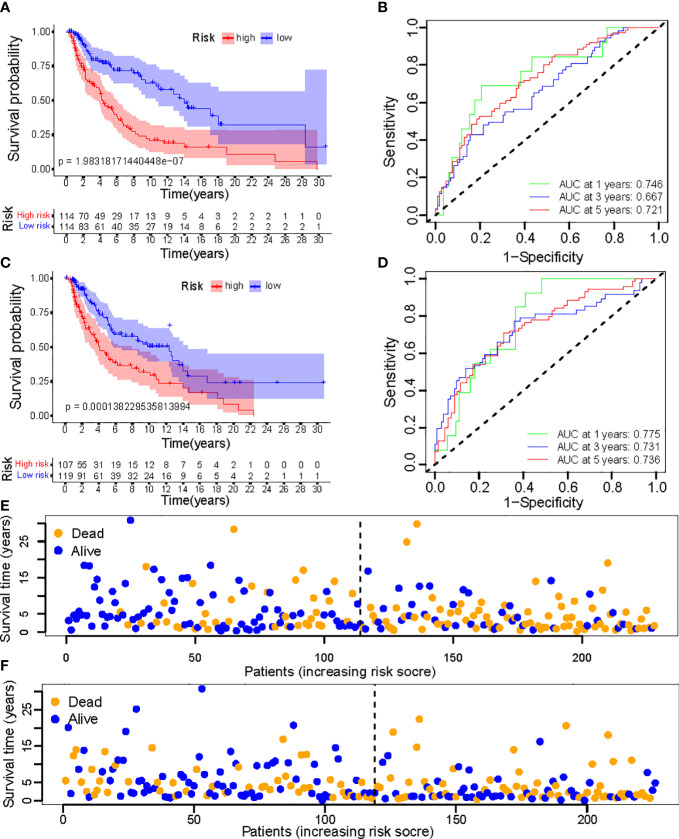
**(A, C)** Kaplan–Meier survival curves of OS of patients between low- and high-risk groups in the learning and training set; **(B, D)** The calibration curves for 1-, 3-, and 5-year OS; **(E, F)** Distribution of survival status and risk score.

To evaluate the feasibility of utilizing the NALncSig to predict OS in the entire TCGA dataset, we randomly divided the entire cohort into the training (N=228) and testing (N=226) cohorts using the same algorithm and regression coefficient (β). As expected, the K-M survival analysis confirmed what was observed in the entire dataset: CM patients in the LR group had longer OS than those in the HR group in both the training and testing cohorts (*p*<0.0001) (**Figures 9A, C**). The predictive efficacy of the risk score was further evaluated using a ROC curve and the AUC value of 1-, 3-, and 5-year OS was 0.746, 0.667, and 0.721, respectively in the training cohort (**Figure 9B**). At the same time, findings from the testing cohort demonstrated AUC value of 1-, 3-, and 5-year OS was 0.775, 0.731, and 0.736, respectively (**Figure 9D**). In predicting the OS of CM patients, the prognostic NALncSig demonstrated improved overall sensitivity and specificity. HR melanoma patients have shorter survival times, according to the training cohort (**Figure 9E**). The testing cohort revealed the same outcome concurrently (**Figure 9F**).

### 3.7 Immune infiltration characteristics and pathways involved

**Figure 10:**
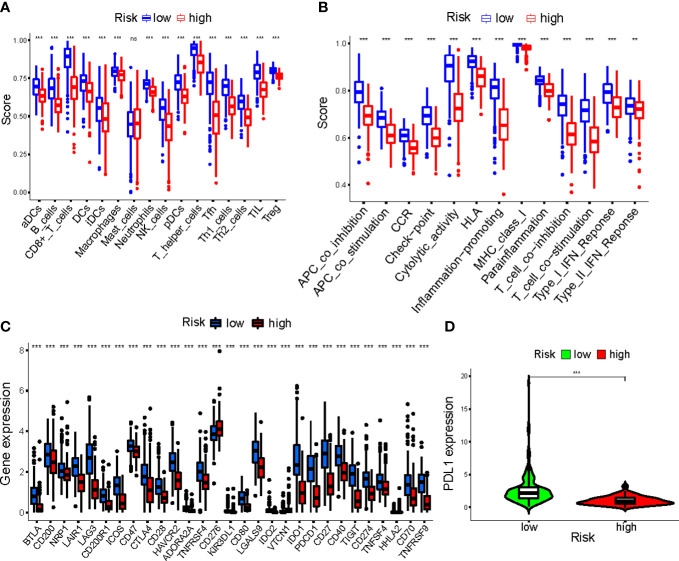
**(A, B)** The ssGSEA scores of immune cells and immune functions in clusters; **(C)** The difference of checkpoints expression in clusters; **(D)** The difference of PDL1 expression in clusters. ***p<0.001. ns, no significance.

**Figure 11:**
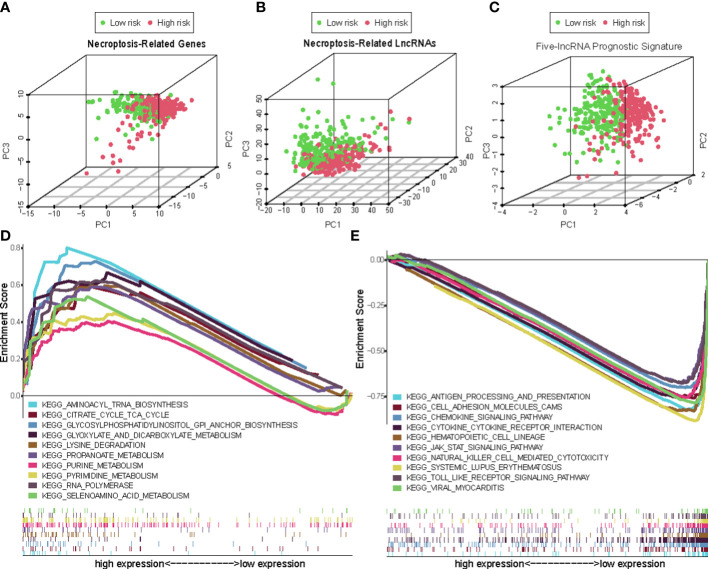
The principal component analysis (PCA) maps show the distribution of patients based on the **(A)** Necroptosis-associated gene sets; **(B)** Necroptosis-associated lncRNAs sets; **(C)** The five-lncRNA prognostic signature; **(D, E)** GSEA of high- and low- risk groups.

Overall, 15 immune cells, including CD8^+^ T cells, dendritic cells (DCs), natural killer cells (NK cells), macrophages, and almost all immunological activity were more strongly activated in the LR group (**Figures 10A, B**). Almost all the immune checkpoints expressed more activity in the LR group, such as CTLA4, and CD274 (programmed cell death-1, ligand 1, PD-L1) (**Figure 10C**). We observed that the LR group had increased PDL1 expression (**Figure 10D**). Principal component analysis (PCA) maps were also used to display the distribution of patients according to necroptosis-associated genes, necroptosis-associated lncRNAs, and five-lncRNA prognostic signature. The results showed that the five-lncRNA prognostic signature was more suitable to distinguish the risk categories of CM patients (**Figures 11A-C**).

### 3.8 Identification of GSEA-derived NALncSig

Using GSEA, we compared the two risk groups to determine which biological processes were highly enriched in one over the other. The samples in the HR group have higher levels of glycosylphosphatidylinositol (GPI)-anchor biosynthesis, cell TCA cycle, and aminoacyl-tRNA biosynthesis. While the samples from the group in the LR group are richer in antigen processing and presentation, cell adhesion, and chemokine signaling (**Figures 11D, E**).

### 3.9 Validation of the expression of necroptosis-associated lncRNA in cell lines

**Figure 12:**
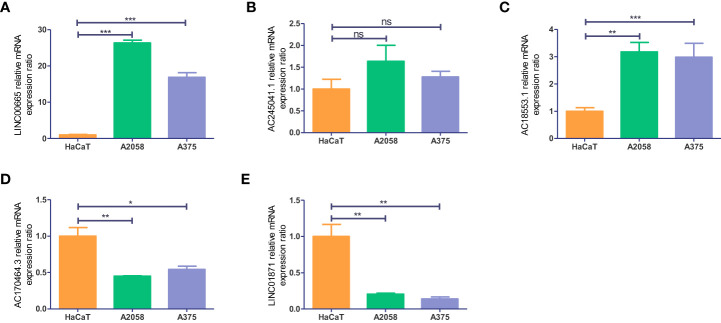
Validation of the expression level of the five necroptosis-associated lncRNAs in cell lines. Expression analysis of **(A)** LINC00665; **(B)** AC245041.1; **(C)** AC018553.1; **(D)** AC107464.3 **(E)** LINC01871. *p<0.05, **p<0.005,***p<0.001. ns, no significance.

To get a further assessment for the expression of necroptosis-associated lncRNA in CM, we selected two melanoma cell lines (A2058 and A375) and a normal keratinocyte cell line (HaCaT) to compare the lncRNAs’ expression. The expression of LINC00665 and AC018553.1 in both melanoma cell lines are higher than that in HaCaT (**Figures 12A, C**). While the expression of LINC01871 and AC107464.3 have both low-expression in A2058 and A375 than HaCaT (**Figures 12D, E**). However, the expression of AC245041.1 showed no statistical significance (**Figure 12B**).

## 4 Discussion

Melanoma mortality has steadily increased over the last few decades, becoming one of the most dangerous types of human cancer (15, 16). As a result, advancements in the molecular characterization and stratification of CM are critical for achieving advances in the disease’s treatment. Finding potential prognostic biomarkers is essential. Necroptosis has a significant role in melanoma invasion, migration, and metastasis since it is implicated in several key ways including the upregulation of death receptors and activation of caspase-8, and mitochondrial complex I inhibition (17, 18) Most studies have focused on the effects of necroptosis on tumor development, treatment resistance, and metastasis; instead, few studies have investigated the potential predictive usefulness of lncRNAs associated with necroptosis in cancer, especially melanoma (19–23).

In this study, a total of 454 patients with CM were randomly divided into a training group, a testing group, as well as a combined group. We summarized 201 necroptosis-associated genes from GeneCards and previous literature. In the combined cohort, we analyzed genes that were differently expressed in GTEx and TCGA. To construct a novel prognostic model, we used uni-Cox analysis and Lasso regression to narrow down the pool of candidate lncRNAs to five (AC245041.1, LINC00665, AC018553.1, LINC01871, and AC107464.3). And these lncRNAs are experimentally verified by qRT-PCR. In these groups, the patients in the HR group had a shorter OS than those in the LR group. Subgroups based on pathological types, grades, M, and N were all shown to be of comparable importance in the prognostic analysis. The multi-Cox regression model identified the necroptosis-associated lncRNA signature as a significant independent risk factor for CM prognosis. A nomogram map was constructed to predict the survival of CM patients at 1, 3, and 5 years. The risk scores are the representation of the clinical outcomes. There was a poor prognosis for patients who scored highly on the necroptosis-associated lncRNAs signature. Also, compared with previously established signature, our signature has a better performance in AUC value (24, 25). ssGSEA showed that the LR group had more immune cell infiltration and more immune functions performing than the HR group. When looking at patients’ immune systems, the principal component analysis revealed that the five-lncRNA prognostic signature varied by patient. PCA showed that the five-lncRNA prognostic signature could differentiate patients according to their immune status. These findings support the use of necroptosis-associated lncRNA as a prognostic signature for CM, particularly when compared to the predictive ability to exist signatures.

Among these five lncRNAs, LINC01871 and AC107464.3 are protective factors, while AC245041.1, AC018553.1, and LINC01871 are risk factors. And these findings were experimentally verified by qRT-PCR. These lncRNAs are widely studied in different types of cancer. For example, AC245041.1 has been certificated to participate in angiogenesis, cell adhesion, wound healing, and extracellular matrix organization processes in stomach adenocarcinoma (26). By regulating the miR-224-5p/VMA21 axis, LINC00665 promotes melanoma cell growth and migration (27). Similarly, silencing LINC00665 inhibits cutaneous melanoma progression *via* the miR-339-3p/TUBB axis (28). AC018553.1 has been identified as a biomarker in melanoma (29, 30). Meanwhile, AC107464.3 has been associated with a lower risk of developing breast cancer (31). LINC01871 has been proven to be a prognostic factor in breast cancer associated with necroptosis (32), autophagy(33), and ferroptosis (34). As was mentioned before, these lncRNAs play significant roles in different cancers to induce either anti- or pro-tumor effects which is consistent with our findings. Consequently, lncRNA-targeted therapy holds a great deal of potential.

The efficiency of immunological functions is greatly influenced by immune cells(35, 36). Our ssGSEA results show the proportion of the infiltrating immune cells in groups. We found that most immune cells are remarkably higher in the LR risk group, such as CD8^+^ T cells, DCs, NK cells, and macrophages. Based on single-cell studies, Sukumar et al. shows melanoma reactivity is linked to a high level of dysfunctionality in CD8^+^ T cells. A rise in CD8^+^ T cell memory, on the other hand, is associated with anti-melanoma effects (37). CD8^+^ T cells release perforin and granules to induce melanoma cell apoptosis (38). In previous studies, CD103^+^ DCs are proven to be critical tumor-draining antigen-presenting cells driving CD8^+^ T cells to elicit strong T cell response in melanoma (39, 40). NK cells exert cytotoxic activity, facilitating the eradication of melanoma (41, 42). Moreover, macrophage activation leads to the phagocytosis of apoptotic or dead melanoma cells or debris (43). Different immune functions collaborate to maintain the immune balance of the tumor microenvironment. Our result shows that HLA, MHC class I, check-point, APC co-inhibition and co-stimulation activities are enhanced. There is evidence that in melanoma, a lack of HLA expression and downregulation of MHC molecules causes T cells to evade immune recognition and subsequently leads to reduced infiltration, which suggests a poor prognosis for the patients (44). It has also been demonstrated that co-stimulatory molecules enhance CD8^+^ tumor-infiltrating lymphocyte expansion and also effector-memory (45, 46). The precise balance between costimulatory and coinhibitory signals determines how effectively the immune system responds. These findings suggest that our conclusion aligns with earlier research suggesting different levels of immune cell infiltration, which leads to melanoma of varying malignancy.

The immunological checkpoint typically has a detrimental effect on immune system control, which is essential for preserving self-tolerance (47). However, tumor cells frequently alter the immunological checkpoint in the tumor, which prevents the immune system from acting as effectively as it could against the tumor (48). Our results showed that checkpoints’ expression is relatively high in the LR group, such as B- and T-lymphocyte attenuator (BTLA), Cytotoxic T lymphocyte associate protein-4 (CTLA4), and programmed cell death protein 1(PD-1). The application of anti-CTLA-4 antibodies like Ipilimumab, and anti-programmed cell death 1 (PD-1) antibody-like Nivolumab have led to a long-term disease control in melanoma patients (49). Programmed cell death-ligand 1 (PDL-1), which is the ligand of PD-1, delivers the inhibitory signals together with PD-1 (50). PDL-1 signals present fresh drug development targets and have the potential to be accurate treatment response indicators in melanoma. Our findings show that the LR group expresses more immune checkpoint blockade-related genes than the HR group, which may cause self-destruction and apoptosis of tumor cells. This result is consistent with previous studies (3, 51). The distinct risk score groupings in this prognostic signature could result in a variable potential for immune treatment, which is crucial in practical application that the LR group receive a better outcome by immunotherapy. In particular, the treatment targeting PDL-1 can achieve a good result and is a priority for future research.

According to earlier research, the immune system changed during melanomagenesis and reduced anti-tumor immunity (3). This study’s GSEA enrichment analysis found that the HR cohort was enriched for aminoacyl tRNA biosynthesis and citrate cycle TCA cycle, while the LR cohort was enriched for antigen processing and presentation and cell adhesion, which may impede immune escape and metastasis. A previous study demonstrated that defects in antigen presentation can predict outcomes to immune checkpoint blockade in melanoma (52). Also, an intact MHC class II antigen presentation pathway improves survival in melanoma(53). The above may be the reason for the better prognosis for the LR group. Meanwhile, it has been discovered that melanoma growth and immune response are influenced by metabolic regulation and metabolic interactions between cancer cells and the microenvironment (54, 55), which was consistent with our result that the HR group is enriched in biosynthesis and metabolism, which may help the proliferation and metastasis of CM.

However, the model we developed still had certain weaknesses despite our attempts to address them using several different approaches. There were problems with the research that was inevitable given that it was conducted in hindsight. Additionally, further research is needed that integrates biochemical tests with clinical prognostic information to properly identify how these lncRNAs impact the prognosis of CM patients through necroptosis.

In conclusion, a signature of five necroptosis-associated lncRNAs was created in this investigation to predict the prognosis of CM. Our results also indicated that NALncSig is a separate risk factor for CM. We hope to provide a new reference for the current prognostic assessment of CM and to shed new light on treatment strategies.

## Data availability statement

The original contributions presented in the study are included in the article/**Supplementary Material**. Further inquiries can be directed to the corresponding authors.

## Author contributions

Conception and design: ZWC and ZL; acquisition, analysis, or interpretation of data: GC, BYL, and YNG; drafting the work: ZWC, BYS, and YHZ; revising the work: JGM and BQS. All authors contributed to the article and approved the submitted version.
