# Supplementary material for: Machine learning-based signature of necrosis-associated lncRNAs for prognostic and immunotherapy response prediction in cutaneous melanoma and tumor immune landscape characterization
Source: Front Endocrinol (Lausanne). 2023 May 9;14:1180732. doi: 10.3389/fendo.2023.1180732 (PMC10203625; doi:10.3389/fendo.2023.1180732)
Supplement: Supplementary file 3 [file Table_3.docx]

Table S3. lncRNA primer sequence

| Gene name | Sequence (5’-3’) |
| --- | --- |
| LINC00665-F | AGGTGCAAAGTGGGAAGTGTG |
| LINC00665-R | GACGCAAAAGGCCAGGACTC |
| AC245041.1-F | AGGGCACTGTCTTTAACGG |
| AC245041.1-R | CAGACACAGGCGTCCAGAAT |
| LINC01871-F | ATTTCTGCTGATGTGTGGTGC |
| LINC01871-R | TTCGGCCTTTGGTAGTGTGA |
| AC107464.3-F | AGGGCTAATTCACATGCCCG |
| AC107464.3-R | GTGTGGGGATGATGGCACG |
| AC018553.1-F | TTACACCCGCTAAAGGCCAG |
| AC018553.1-R | CCAGCAGAAAGGGCAACAAC |
